# Diagnostic and Therapeutic Considerations for Oncology Patients With *Clostridium difficile* Infection

**DOI:** 10.1200/JOP.2016.019141

**Published:** 2016-12-16

**Authors:** Michael H. Woodworth, Colleen S. Kraft

**Affiliations:** Emory University, Atlanta, GA

Neeman and Freifeld^1^ review important aspects of care for *Clostridium difficile* infection in oncology patients, a group with specific risk factors for this disease that are not commonly addressed in other reviews. They summarize aspects of epidemiology, diagnosis, treatment, and future directions to improve care for these patients.

## Diagnosis and Classification

Despite the impressive burden of *C difficile* infection in the United States, it has become clear that there are many subtle complexities in diagnosis and classification of *C difficile* infection, which is also the case in oncology patients. Although toxigenic culture for *C difficile* is the gold-standard diagnostic technique, this method is infrequently performed because of its complex laboratory protocol requiring experienced technologists, delayed turnaround time for results, and limited scalability. Nucleic acid amplification tests are highly sensitive but have correspondingly high rates of false-positive results.^2^ Even a test with 99% sensitivity and 99% specificity (or combination test algorithms with similar performance characteristics) will produce false-positive results. As Neeman and Freifeld^1^ point out, overdiagnosis of *C difficile* infection in these patients puts them at risk for overtreatment. Overtreatment, in turn, worsens the risk of intestinal dysbiosis, defined as abnormal intestinal microbiota composition or lack of diversity. Oncology patients are already at high risk of developing intestinal dysbiosis through frequent and prolonged health care exposures, and providers should be mindful of other factors that may exacerbate this risk. As examples, in a mouse model, metronidazole was shown to have prolonged detrimental changes in gut microbiota composition, and in an industry-supported study, use of enteric vancomycin was shown to increase risk of colonization with *Candida* and vancomycin-resistant *Enterococcus*.^3,4^ Additional work is needed to better understand ideal tests and frequency of *C difficile* testing in oncology patients.

There are multiple scoring systems for severity of *C difficile* infection, but the importance of classification of infection severity should depend on the implications for clinical care. It is not clear that such classification improves outcomes if therapies such as oral vancomycin or fecal microbiota transplantation are increasingly considered as first-line treatments. The potential to introduce collider bias is another challenge of such scoring systems when they include multiple factors related to *C difficile* infection that could be either causes or effects (eg, intensive care unit admission or hospitalization) rather than causal factors alone. Collider bias is the creation of an artificial statistical relationship between two factors when conditioning on common effects of a disease rather than common causes alone.^5^ The 2010 Infectious Diseases Society of America clinical practice guidelines for *C difficile* identified appropriate treatment of *C difficile* infection by severity category as a research gap for future study.^6^ Unfortunately, there has been little update in the state of the literature on this topic since the release of these guidelines. Remaining questions about the clinical importance of severity classification and validation of these scores warrant further study.

## Microbial Treatment of Infection and Dysbiosis

Our group has particular interest in the use of microbial-based therapeutics for immunocompromised patients. Although these patients are certainly at increased risk of infection as a result of their underlying diseases and treatments, administration of carefully screened and processed microbial material may actually reduce risk of such infections if the material has a more diverse composition that resembles that of healthy individuals. We are encouraged by the growing number of registered clinical trials on ClinicalTrials.gov for fecal microbiota transplantation. Many of these studies are phase I trials investigating the safety of fecal microbiota transplantation and other microbial-based therapeutics in immunocompromised patients. We are watching for the results of these studies with interest and anticipate that they will have an important impact for oncology patients in particular and immunocompromised patients more generally.

## Future Directions

In many centers, oncology care is delivered by specialized teams comprising nurses, providers, and pharmacists on dedicated units. Such organization of care affords important opportunities for antibiotic stewardship to reduce *C difficile* infection and infections with other multiple drug–resistant organisms.^7^ In the near term, we are also likely to see large changes in practice in screening for toxigenic *C difficile* in asymptomatic carriers, because this was recently shown to greatly reduce subsequent rates of infection.^8^

*C difficile* infection is likely to remain a leading cause of nosocomial infection as long as broad-spectrum antibiotics are widely used as an initial approach to treatment of infectious diseases without a widely available rescue for antibiotic-induced dysbiosis. Special attention to oncology patients with *C difficile* infection will improve understanding and care of these patients with unique risk factors for this disease.
